# Anthraquinones as Potential Antibiofilm Agents Against Methicillin-Resistant *Staphylococcus aureus*

**DOI:** 10.3389/fmicb.2021.709826

**Published:** 2021-09-03

**Authors:** Zhi-Man Song, Jun-Liang Zhang, Kun Zhou, Lu-Ming Yue, Yu Zhang, Chang-Yun Wang, Kai-Ling Wang, Ying Xu

**Affiliations:** ^1^Shenzhen Key Laboratory of Marine Bioresource and Eco-Environmental Science, Shenzhen Engineering Laboratory for Marine Algal Biotechnology, College of Life Sciences and Oceanography, Shenzhen University, Shenzhen, China; ^2^Department of Chemistry, The University of Hong Kong, Pokfulam, Hong Kong, China; ^3^College of Pharmacy, Institute of Materia Medica, Dali University, Dali, China; ^4^Key Laboratory of Marine Drugs, The Ministry of Education of China, School of Medicine and Pharmacy, Ocean University of China, Qingdao, China; ^5^Laboratory for Marine Drugs and Bioproducts, Qingdao National Laboratory for Marine Science and Technology, Qingdao, China; ^6^Institute of Evolution and Marine Biodiversity, Ocean University of China, Qingdao, China

**Keywords:** anthraquinones, antibiofilm agents, *Kitasatospora albolonga*, RNA-Seq, antibiotics, Pst system

## Abstract

Biofilms formed by methicillin-resistant *Staphylococcus aureus* (MRSA) are one of the contributing factors to recurrent nosocomial infection in humans. There is currently no specific treatment targeting on biofilms in clinical trials approved by FDA, and antibiotics remain the primary therapeutic strategy. In this study, two anthraquinone compounds isolated from a rare actinobacterial strain *Kitasatospora albolonga* R62, 3,8-dihydroxy-l-methylanthraquinon-2-carboxylic acid (**1**) and 3,6,8-trihydroxy-1-methylanthraquinone-2-carboxylic acid (**2**), together with their 10 commercial analogs **3**–**12** were evaluated for antibacterial and antibiofilm activities against MRSA, which led to the discovery of two potential antibiofilm anthraquinone compounds anthraquinone-2-carboxlic acid (**6**) and rhein (**12**). The structure-activity relationship analysis of these anthraquinones indicated that the hydroxyl group at the C-2 position of the anthraquinone skeleton played an important role in inhibiting biofilm formation at high concentrations, while the carboxyl group at the same C-2 position had a great influence on the antibacterial activity and biofilm eradication activity. The results of crystal violet and methyl thiazolyl tetrazolium staining assays, as well as scanning electron microscope and confocal scanning laser microscopy imaging of compounds **6** and **12** treatment groups showed that both compounds could disrupt preformed MRSA biofilms possibly by killing or dispersing biofilm cells. RNA-Seq was subsequently used for the preliminary elucidation of the mechanism of biofilm eradication, and the results showed upregulation of phosphate transport-related genes in the overlapping differentially expressed genes of both compound treatment groups. Herein, we propose that anthraquinone compounds **6** and **12** could be considered promising candidates for the development of antibiofilm agents.

## Introduction

Bacterial biofilms are surface or interphase-attached microbial communities that are encapsulated in self-secreted extracellular matrix comprising largely of proteins, polysaccharides, nucleic acids, and lipids (Costerton et al., 1999; Flemming and Wingender, 2010). Bacteria growing inside biofilms are more extremely resistant to hostile environment, antimicrobial agents, and mechanical stresses than their planktonic counterparts (Gilbert et al., 2002; Fux et al., 2005; Hoiby et al., 2010, 2011). It is reported that more than 80% of chronic infections are related with the formation of biofilms by pathogens and very difficult to tackle (Potera, 1999; Lebeaux et al., 2013).

Antimicrobial resistance of *Staphylococcus aureus* has become a global health threat. As a notorious member of the drug-resistant *S. aureus* family, methicillin-resistant *S. aureus* (MRSA) causes various diseases, ranging from minor skin infections to fatal necrotizing pneumonias (Boyce et al., 2005; Calo et al., 2007; DeLeo et al., 2010). Conventional antibiotics are becoming ineffective in the treatment of biofilm-forming MRSA and their biofilms (Laplante and Mermel, 2009; Kelley et al., 2011; Gu et al., 2013; Hall Snyder et al., 2014; Meeker et al., 2016). There is an urgent need to explore new antibiofilm agents that prevent the formation of MRSA biofilms and/or disrupt the preformed biofilms (Brady et al., 2011; Craigen et al., 2011; Kaplan et al., 2012; Ranall et al., 2012; Bjarnsholt et al., 2013; Alves et al., 2014; Bhattacharya et al., 2015).

Natural products cannot be ignored as a potential source for the exploitation of bioactive substances (Gunatilaka, 2006; Li and Vederas, 2009; Ranall et al., 2012; Fletcher et al., 2014). Some natural compounds isolated from plants [resveratrol (Augustine et al., 2014), quercetin (Lee et al., 2013), magnolol (Wang et al., 2011), baicalein (Zeng et al., 2008), ellagic acid (Quave et al., 2012), phloretin (Lee et al., 2011), oroidin (Richards et al., 2008), etc.] and microbes [4-phenylbutanoic acid (Nithya et al., 2011), glycolipid (Dusane et al., 2011), butanolide (Yin et al., 2019), etc.] have been reported to exhibit excellent antibiofilm activity (Gunatilaka, 2006; Wang et al., 2011). Anthraquinones (AQs), as a big family of natural products, have been demonstrated to have antibacterial, antibiofilm, anti-inflammatory, antioxidative, anticancer, and antiviral activities (Barnard et al., 1992; Galasinski et al., 1996; Agarwal et al., 2000; Yen et al., 2000; Bashir et al., 2011; Lin et al., 2015; Nam et al., 2017). Among them, four AQs have been proved to be able to reduce biofilm formation efficiently (Coenye et al., 2007; Lee et al., 2016; Farooq et al., 2017; Manoharan et al., 2017). Two alkyl-substituted anthraquinone derivatives, symploquinones A and C, inhibited the biofilm formation of *Streptococcus mutans*, *S. aureus*, and *Proteus mirabilis* with low minimal inhibitory concentration (MICs) (83–160 μg/ml) (Farooq et al., 2017). Emodin could significantly reduce biofilm formation of *S. mutans* and *S. aureus* (Coenye et al., 2007) and alizarin at 10 μg/ml inhibited biofilm formation of *S. aureus* significantly (Lee et al., 2016). These studies suggested that some AQs could be considered promising inhibitors of biofilms. Unfortunately, most of them are only capable of inhibiting initial biofilm formation instead of eradicating existing biofilms, although the latter is more relevant in clinical infection. Until now, to our best knowledge, biofilm eradication function of AQs has not been reported yet.

In this study, two known natural anthraquinone compounds 3,8-dihydroxy-1-methylanthraquinone-2-carboxylic acid (**1**) and 3,6,8-trihydroxy-1- methylanthra-quinone-2-carboxylic acid (**2**) from the actinomycete *Kitasatospora albolonga* R62, together with their 10 commercial analogs **3**–**12** were screened for antibacterial and antibiofilm activity against MRSA, and then analyzed for their structure-activity relationships (SARs). It was found that anthraquinone-2-carboxlic acid (**6**) and rhein (**12**) could effectively eradicate biofilms formed by MRSA strain ATCC43300. Stanning method [crystal violet (CV) and methyl thiazolyl tetrazolium (MTT)] and microscopic technology [scanning electron microscope (SEM) and confocal scanning laser microscopy (CLSM)] were employed to determine the potential eradication activity of these two compounds against the preformed MRSA biofilms. Based on transcriptome profiling and quantitative reverse transcription polymerase chain reaction (qRT-PCR) validation, we propose that the antibiofilm mechanism of compounds **6** and **12** might involve dispersing biofilm cells and upregulation of phosphate transport-related genes.

## Materials and Methods

### Sample Collection and Bacterial Identification

The soil sample was collected from the rhizosphere sediment of the mangrove plant *Kandelia candel* (L.) Druce in Mai Po Nature Reserve, Hong Kong (114.05°E, 22.49°N). Strain R62 was isolated from the sample through stepwise purification. The 16S rRNA gene of strain R62 was amplified by two universal primers of 27F (5′-AGAGTTTGATCMTGGCTCAG-3′) and 1492R (5′-TACGGYTACCTTGTTACGACTT-3′). The 16S rRNA gene sequence displayed 100% similarity with *K. albolonga* NBRC 13465.

### Fermentation, Extraction, and Compound Isolation

The actinobacterial strain *K. albolonga* R62 was distributed to 250 ml Erlenmeyer flask containing 80 ml SGTYP medium (5 g of starch, 5 g of glucose, 1 g of peptone, 1 g of tryptone, 1 g of yeast extract, and 17 g artificial sea salt is dissolved in 1 L double-distilled water, pH 7.4–7.6) and fermented at 28°C, 200 rpm for 5 days. In total, nearly 15 L of culture broth was centrifuged at 10,000 × *g* for 15 min to remove cells. The supernatant was extracted using ethyl acetate (ν/ν 1:3) three times to yield an EtOAc extract (3.0 g) after concentration. The extract was separated by ODS open column elution with a gradient of MeOH-H_2_O (20–100%, at intervals of 10%) to afford nine fractions (Fr. 1–Fr. 9). HPLC-MS analysis of these fractions showed that natural products are mainly concentrated in Fr. 3 and Fr. 4. Both fractions were isolated and purified by semipreparative HPLC (Waters, Parsippany, NJ, United States) using semipreparative reverse-phase Phenomenex C18 columns (5 μm, 250 × 10 mm in size) with a gradient mobile phase of ACN-H_2_O (40–60%) to yield compounds **1** and **2**.

### Experimental Strain and Compounds

**FIGURE 1:**
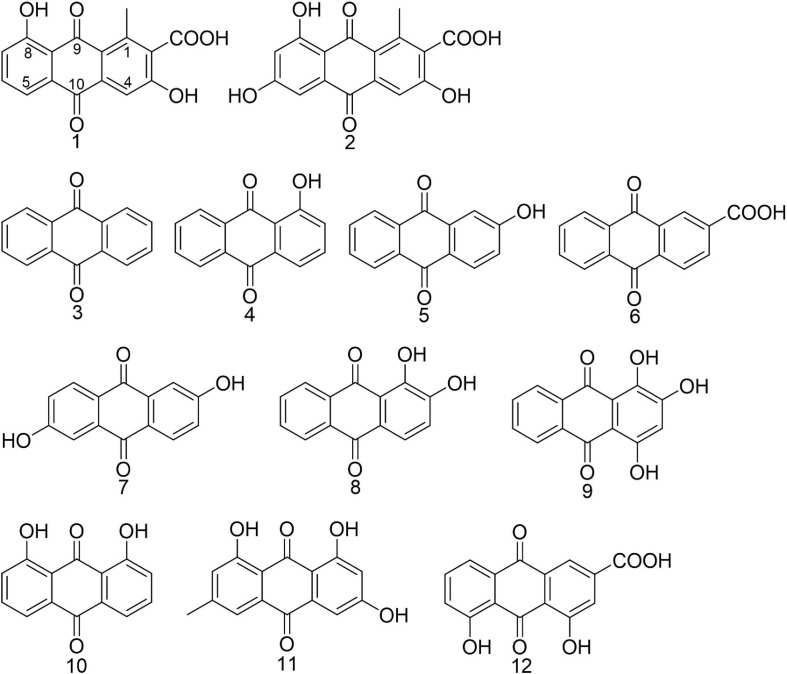
Chemical structures of anthraquinones mentioned in the article. Compounds **1** and **2** were isolated from *Kitasatospora albolonga* R62 and compounds **3**–**12** were commercially obtained.

Methicillin-resistant *Staphylococcus aureus* strain ATCC 43300 was used as biofilm model organism. Anthraquinone (**3**, MCKLIN, China), 1-hyrdroxyanthraquanone (**4**, Yuanye Biotechnology, China), 2-hydroxyanthraquanone (**5**, Yuanye Biotechnology, China), anthraquinone-2-carboxlic acid (**6**, Sigma-Aldrich, Japan), 2,6-dihydroxyanthraquinone (**7**, Energy Chemical, China), alizarine (**8**, Aladdin, China), purpurin (**9**, Solarbio, China), 1,8-dihydroxyanthraquinone (**10**, Aladdin, China), emodin (**11**, Aladdin, China), and rhein (**12**, MCKLIN, China) were all commercially available. All compounds tested were dissolved in DMSO to get the stock solution. The structure of commercially obtained AQs is shown in Figure 1, and their LC-MS data are shown in Supplementary Figure 1.

### The Determination of Minimum Inhibitory Concentration

The minimum inhibitory concentrations (MICs) of AQs were determined according to the Clinical and Laboratory Standards Institute (CLSI) guideline. The experiment was conducted in 96-well microplates (Corning, Corning, NY, United States). In brief, a stock solution of 20 μg/μl of each compound was prepared in DMSO, diluted 100 times with bacterial culture (∼5 × 10^5^ CFU/ml) to 200 μg/ml, and further diluted by a serial twofold dilution to obtain the tested solutions with five concentrations ranging from 100 to 6.25 μg/ml. One microliter of DMSO was used as the negative control. Cell growth was monitored by the absorbance at OD_595_ using the spectrophotometer (Varioskan Flash, Thermo Scientific, Waltham, MA, United States).

### The Evaluation of Inhibitory Activity Against MRSA Biofilm Formation

The inhibition of static biofilm formation was tested as previously described (Stepanovic et al., 2007; Nair et al., 2016; Yin et al., 2019). After overnight cultivation, 100 μl/well of the bacterial culture, diluted in LB broth with 0.5% glucose (about 5 × 10^6^ CFU/ml), was aliquoted into 96-well microplates (Corning, United States) with 1 μl of different concentrations (20 or 5 μg/μl) of each compound or DMSO and incubated at 37°C for 24 h. Using the same method, 1 ml/well of the diluted culture was added into 24-well microplates (Corning, United States) with 2 μl of compounds (100 or 25 μg/μl) or DMSO. After incubation, each well was rinsed with 1 × PBS to remove non-adherent cells and then dried at 37°C. CV staining was used to determine the remaining total biofilm biomass. Biofilms were stained with 0.5% CV for 10 min before washing. The washed biofilm mass was then dissolved in 30% acetic acid, and then the absorbance was measured at 550 nm.

### Biofilm Eradication Assay

The 24-h mature biofilms containing no compounds or DMSO preformed in 24-well microplates according to the described method (Nair et al., 2016; Yin et al., 2019). Each well was rinsed twice by 1 × PBS, followed by adding fresh LB broth with 0.5% glucose and 2 μl of different concentrations (25 and 100 μg/ml) of each compound or DMSO, respectively, then incubated at 37°C for another 24 h. The rest of the washing and CV staining steps were the same as the static biofilm formation assay. Additionally, 3-(4,5-dimethyl-2-thiazolyl)-2,5-diphenyl-2H-tetrazoliumbromide (MTT; HiMedia, West Chester, PA, United States) staining was conducted to show the number of viable cells in the remaining biofilms at 550 nm. Both CV staining and MTT staining assay were used to determine the eradication assay of compounds **6** and **12** in the gradient-diluted concentrations (6.25, 12.5, 25, 50, and 100 μg/ml).

### Biofilm Eradication Imaging

Bacterial biofilms were grown on the glass coverslips which were at the bottom of 24-well microplates at the beginning. The biofilm eradication assay was then carried out according to the method described in Yin et al. (2019) and Nair et al. (2016). For SEM imaging (Nithya et al., 2010; Sultana et al., 2016; Yin et al., 2019), before drying, the biofilms were fixed by 2.5% glutaraldehyde in 0.1 M phosphate for 24 h, followed by rinsing with 1 × PBS and dehydrated by a gradient of ethanol (50, 70, 80, 90, 95, and 100% ethanol for 10 min each). The biofilms were then subjected to analysis using a SEM (SU-70, HITACHI, Japan) after air drying and sputter coating with gold (50 s). The CSLM (TCS SP8, Leica, Germany) analysis was implemented as describe by the reported articles (Nithya et al., 2010; Dusane et al., 2011; Yin et al., 2019). In brief, the biofilms were washed with 1 × PBS after 24 h of treatment and dried at 37°C for 30 min, then stained with the LIVE/DEAD *Bac*Light Bacterial Viability Kit (L7007, Invitrogen, Carlsbad, CA, United States) in the dark. The biofilms were observed using a ×20 objective, and images were acquired with 1,024 × 1,024 resolutions. The CLSM was conducted at 488 and 561 nm.

### RNA Extraction, Library Construction, Sequencing, and Data Analysis

Samples were collected according to the method mentioned in the biofilm eradication assay. Mature MRSA biofilms were treated with compound **6** or **12** at the concentration of 100 μg/ml and washed by 1 × PBS three times after 8 h. The same volume of DMSO-treated mature biofilms was used as control. Total RNA of all the samples was extracted using Trizol reagent (Invitrogen) according to the manufacturer’s instructions. RNA qualification was examined by 1% agarose gel electrophoresis and the NanoDrop spectrophotometer (Thermo Fisher Scientific). Strand-specific libraries were constructed using the TruSeq RNA sample preparation kit (Illumina, San Diego, CA, United States) and sequencing with Illumina nova-seq6000.

The quality of raw data was checked by FastQC version 0.11.2.^1^ High-quality clean reads were mapped to reference gene *S. aureus* A9754 sequence (genome assembly: ASM17799v1) using STAR (2.5.3a) (Dobin et al., 2013). All mapped reads were then annotated by BLASTX against NCBI non-redundant protein database (nr) and Swiss-Prot. Gene IDs of the whole detected sequence were named according to the information on the ensemble bacterial database for analysis.^2^ The fragments per kilobase of transcript per million mapped reads (FPKM) for the mapped sequences of each sample were calculated and normalized by Cuffnorm (Trapnell et al., 2012), and the differential expression of genes between control and compound treatment groups were analyzed with DESeq2 (version 1.16.1) (Love et al., 2014). Some differentially expressed genes (DEGs; *p* < 0.05 and absolute fold change ≥ 2) were chosen for subsequent analysis of gene function and signaling pathway enrichment based on searching against Gene Ontology (GO) and Kyoto Encyclopedia of Genes and Genomes (KEGG) database (Sun et al., 2017; Wang et al., 2018). The GO functional annotation of the DEGs was determined with topGO package (Alexa and Rahnenfuhrer, 2010) for each gene clusters, and the KEGG pathway enrichment analysis was performed using phyper algorithm in R 3.6.1^3^ against the KEGG database. Significantly enriched GO terms and KEGG pathways were selected using a threshold of *p*-value of ≤0.05.

### qRT-PCR

To confirm the RNA-Seq data, the relative expression of some related genes was assessed by qRT-PCR. Total RNA was extracted by the E.Z.N.A.^®^ Bacterial RNA Kit (Omega Bio-Tec, Norcross, GA, United States). cDNA was synthesized using a PrimeScript RT reagent kit with a gDNA Eraser (TaKaRa, Tokyo, Japan). qRT-PCR reactions (20 μl in total volume with 4 μl of cDNA product) were carried out using TaKaRa SYBR Premix Ex Taq (Tli RNaseH Plus) in a CFX96 Real-Time System (BioRad, Hercules, CA, United States). All the primers used in the present study had been listed in the Supplementary Table 1. Housekeeping gene *pyk* was used as an internal reference to normalize the qRT-PCR date (Tan et al., 2015). Three independent replicates with four replications were tested in each qRT-PCR run.

### Statistical Analysis

All the experiments were performed in at least three independent experiments and three biological replicates each time. Single-factor analysis of variances followed with Ryan-Einot-Gabriel-Welsch *F*-test as the *post hoc* method were performed to test the differences of the antibiofilm activity between 12 different compounds using SPSS software 16.0 in Figure 4. One-way ANOVA and Multiple *t*-tests in GraphPad Prism 7.0 were performed to check the other statistical comparisons in Figures 5, 11 (*p* < 0.001 and 0.01).

## Results

### Identification of Compounds **1** and **2**

Compound **1** (2.5 mg), orange powder, was identified as 3,8-dihydroxyl-methylanthraquinon-2-carboxylic acid (DMCA; Figure 1) by the NMR data and LC-MS data (Supplementary Figures 2–4) according to the reported data (Krupa et al., 1989). ESI-MS m/z: 299.055 [M+H]^+^ (calculated for C_16_H_10_O_6_ 299.055), 297.041 [M−H]^–^ (calculated, 297.040). ^1^H NMR (600 MHz, *d*_6_-DMSO) δ: 12.90 (s, 1H), 7.72 (t, *J* = 7.9 Hz, 1H), 7.64 (d, *J* = 7.4 Hz, 1H), 7.58 (s, 1H), 7.34 (d, *J* = 8.2 Hz, 1H), 2.71 (s, 3H); ^13^C NMR (DMSO-*d*6, 150 MHz) δ: 189.8, 182.6, 168.7, 161.9, 141.6, 136.8, 136.5, 133.0, 131.3, 124.9, 122.7, 118.8, 117.4, 112.8, and 20.4.

Compound **2** (2.0 mg), orange powder, was identified as 3,6,8-trihydroxy-1-methylanthraquinone-2-carboxylic acid (TMCA; Figure 1) by the NMR data and LC-MS data (Supplementary Figures 5, 6) according to the reported data (Hawas et al., 2006). ESI-MS m/z: 313.036 [M−H]^–^ (calculated, 313.035), 315.050 [M+H]^+^ (calculated, 315.050). ^1^H NMR (600 MHz, *d*_6_-DMSO) δ: 13.14 (s, 1H), 11.11 (s, 1H), 7.58 (s, 1H), 7.07 (d, *J* = 2.4 Hz, 1H), 6.60 (d, *J* = 2.4 Hz, 1H), 2.68 (s, 3H).

### Biofilm Inhibition Activity of Natural Products 1 and 2 at Concentrations Lower Than MIC Values

**FIGURE 2:**
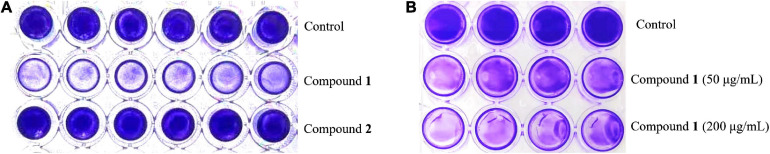
The formation of MRSA biofilms after treatment with compound **1** or **2** staining by CV. The biofilm inhibition activity results after treatment with compound **1** or **2** (200 μg/ml) in a 96-well plate **(A)** and different concentrations of compound **1** (50 and 200 μg/ml) in a 24-well plate **(B)**. The same volume of DMSO was used as negative control.

The preliminary screening results of antibiofilm activities of compounds **1** and **2** revealed that compound **1** had a significant effect on decreasing MRSA biofilm formation by >50% at the concentration of 200 μg/ml while its MIC value was higher than 200 μg/ml (Figure 2), but compound **2** showed no obvious bactericidal activity or antibiofilm activity at the same concentration.

### Antibacterial and Antibiofilm Activities of Anthraquinones

**FIGURE 3:**
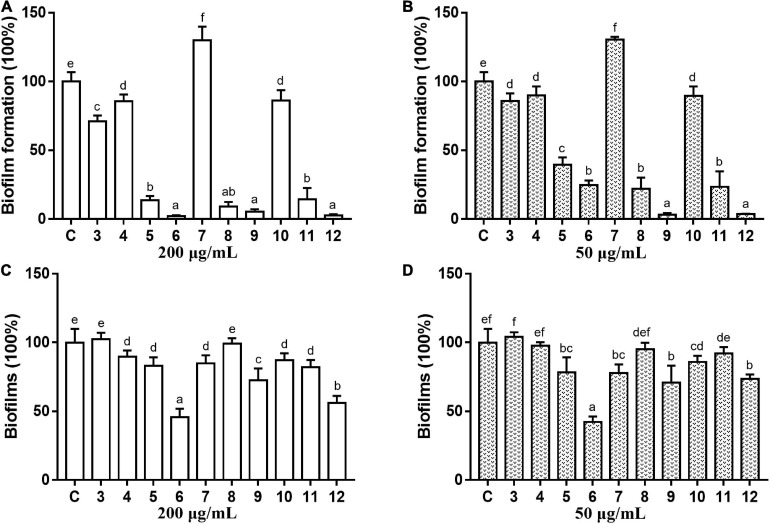
Antibiofilm activity of commercial anthraquinones **3–12** against MRSA. The MRSA biofilm inhibitory activity **(A,B)** and the biofilm eradication activity **(C,D)** of each compound at two different concentrations (**A,C** 200 μg/ml; **B,D** 50 μg/ml). The same volume of DMSO was used as negative control. C, control. Results are presented as percentages of the control group. The data represent mean ± SD (*n* = 9). Means in a group with different letters are significant differences based on Ryan-Einot-Gabriel-Welsch multiple *F*-test, *p* < 0.001.

The antibacterial and antibiofilm activities of the other 10 commercial AQs **3**–**12** (Figure 1) against MRSA ATCC43300 were investigated to analyze their SARs. Among them, compounds **6** and **12** displayed antibacterial activity with MIC values of 100 and 12.5 μg/ml, respectively. The other compounds did not show antimicrobial activity against planktonic MRSA cells at the concentration of 200 μg/ml (Table 1). Meanwhile, antibiofilm activities (including the inhibitory effects on the formation of MRSA biofilms and the eradication abilities against preformed biofilms) of compounds **3**–**12** were evaluated at concentrations of 200 and 50 μg/ml by the CV staining method (Figure 3). Compounds **5**, **6**, **8**, **9**, **11**, and **12** could all strongly inhibit the formation of MRSA biofilms by >50% at the concentration of 50 μg/ml. It is worth noting that the inhibition rates were 75.2% (±3.3%) and 96.4% (±0.3%) for compounds **6** and **12**, respectively. On the contrary, compound **7** promoted biofilm forming which was not parallel to the trend of this class of compounds (Figure 3A). In addition to their good biofilm formation inhibitory activities, compounds **6** and **12** also showed good eradication activity against mature MRSA biofilms. Compound **6** could eradicate 54.2% (±5.9%) and 57.7% (±3.8%) of the mature MRSA biofilms at 200 and 50 μg/ml, and compound **12** could eradicate 44.0% (±3.8%) and 26.5% (±3.2%) at 200 and 50 μg/ml, respectively (Figure 3B). Based on their outstanding performance in both inhibiting and eradicating MRSA biofilms, we therefore chose compounds **6** and **12** as representatives for further antibiofilm study.

**TABLE 1:** Minimum inhibitory concentrations of anthraquinones.

Compound	MIC (μg/ml)	Compound	MIC (μg/ml)
DMCA (**1**)	>200	2,6-Dihydroxyanthraquinone (**7**)	>200
TMCA (**2**)	>200	Alizarin (**8**)	>200
Anthraquinone (**3**)	>200	Purpurin (**9**)	>200
1-Hydroxyanthraquinone (**4**)	>200	1,8-Dihydroxyanthraquinone (**10**)	>200
2-Hydroxyanthraquinone (**5**)	>200	Emodin (**11**)	>200
Anthraquinone-2-carboxlic acid (**6**)	100	Rhein (**12**)	12.5

*DMCA, 3,8-dihydroxy-1-methylanthraquinone-2-carboxylic acid (**1**); TMCA, 3,6,8-trihydroxy-1-methylanthraquinone-2-carboxylic acid (**2**).*

*The bold value behind the compound indicates the compound number corresponding to Figure 1 and the entire article.*

### Biofilm Eradication Activity of Compounds **6** and **12**

**FIGURE 4:**
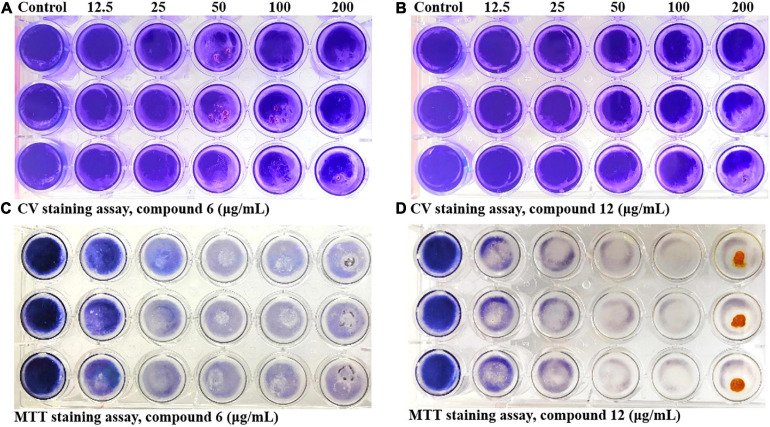
Dose-dependent effects of compounds **6** and **12** against MRSA biofilms qualified by two different staining methods. The 24-h preformed MRSA biofilms were treated with increasing concentrations of compounds **6** and **12** (12.5–200 μg/ml) for another 24 h before staining with CV **(A,B)** or MTT **(C,D)** solution. The same volume of DMSO was used as negative control. At least three independent experiments were conducted, and there were three biological replicates each time.

**FIGURE 5:**
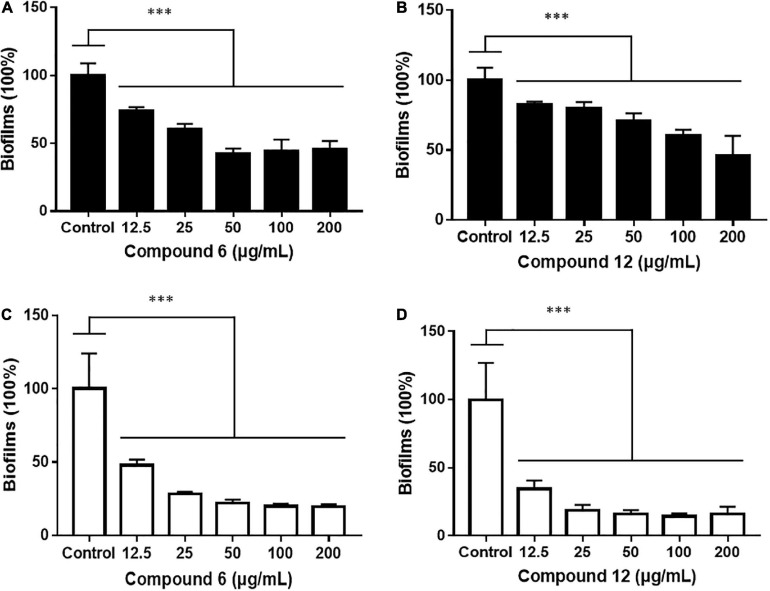
Relative values of dose-response effects of compounds **6** and **12** against MRSA biofilms qualified by two different staining methods. The 24-h preformed biofilms were treated with different concentrations of compounds **6 (A,C)** and **12 (B,D)** using CV staining **(A,B)** and MTT staining **(C,D)** methods. The same volume of DMSO was used as negative control. Results are presented as percentages of the control. The data represent mean ± SD (*n* = 9); ^∗∗∗^*p* < 0.001.

Two kinds of staining methods were conducted to further qualify the dose-dependent effects of compounds **6** and **12** against MRSA biofilms. The remaining total biomass in biofilms was determined by the CV staining method, while the metabolic activity of the viable cells was investigated by MTT staining method. Both compounds displayed only weak to moderate effect on reducing the total biomass of 24 h mature MRSA biofilms at concentrations ranging from 12.5 to 200 μg/ml (Figures 4A,B, 5A,B). However, these two compounds could significantly disperse the living cells inside biofilms and showed strong eradicating efficiency with concentrations ranging from 12.5 to 200 μg/ml (Figures 4C,D, 5C,D). The remaining rates of viable biofilm cells were 47.9% (±3.8%) and 34.5% (±6.1%) at the lowest tested concentration of 12.5 μg/ml, respectively.

### SEM and CLSM Analysis of Biofilm Structures After Treatment With Compounds **6** or **12**

**FIGURE 6:**
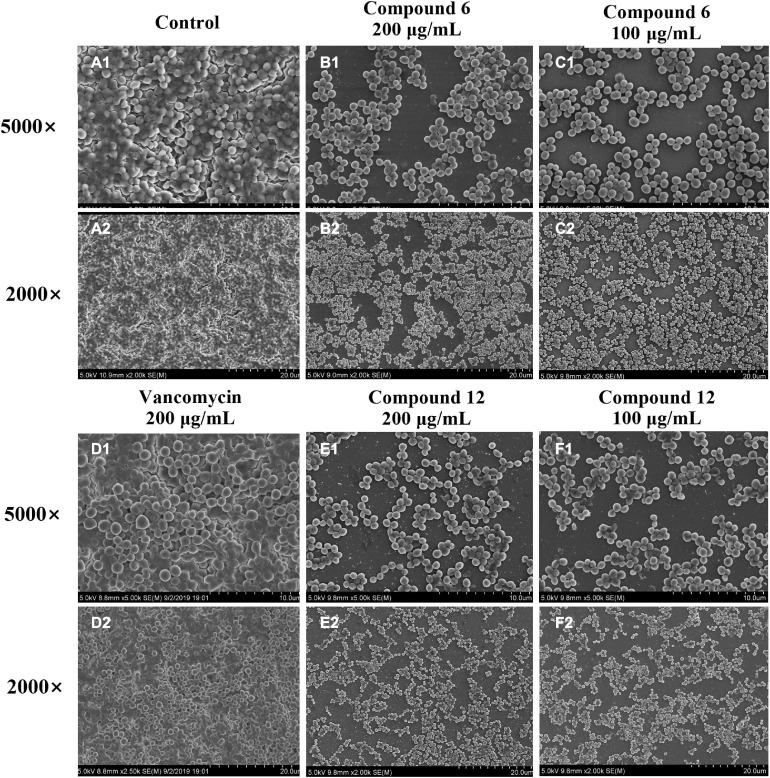
Scanning electron micrographs of 24-h preformed MRSA biofilms treated with different concentrations of compounds **6** and **12**. **(A1–F2)** Different treatment groups (**A1,A2** DMSO control; **B1,B2** 200 μg/ml of compound **6**; **C1,C2** 100 μg/ml of compound **6**; **D1,D2** 200 μg/ml of vancomycin; **E1,E2** 200 μg/ml of compound **12**; **F1,F2** 100 μg/ml of compound **12**). Series 1 and 2 were different magnifications with ×5,000 and ×2,000, respectively.

**FIGURE 7:**
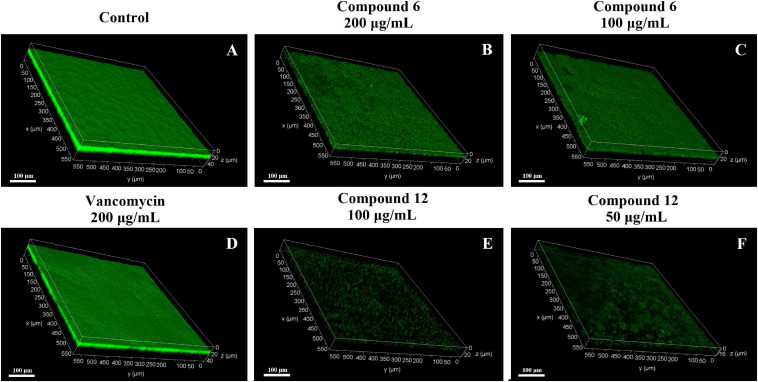
Confocal scanning laser micrographs of 24-h preformed MRSA biofilms treated with different concentrations of compounds **6** and **12**. **(A–F)** Different treatment groups (**A** DMSO control; **B** 200 μg/ml of compound **6**; **C** 100 μg/ml of compound **6**; **D** 200 μg/ml of vancomycin; **E** 100 μg/ml of compound **12**; **F** 50 μg/ml of compound **12**). Scale bars represent 100 μm.

SEM and CLSM were used to analyze the 24 h preformed mature MRSA biofilm structures after treatment with compounds **6** and **12** for another 24 h. The images of SEM showed that the total MRSA biofilm biomass was significantly reduced when treated with compounds **6** and **12** at concentrations of 200 and 100 μg/ml (Figure 6). There were not many differences between compounds **6**- and **12**-treated groups. It also should be noticed that the cell walls and membranes of the biofilm cells after treatment with both compounds remained as intact as those in the negative control group. Meanwhile, the viable bacteria within biofilms could be represented by the thickness and fluorescence intensity of the three-dimensional structures of MRSA biofilms in CLSM images (Figure 7), which showed that compound **12** at 50 and 100 μg/ml was highly effective in removing viable cells, and compound **6** at 100 and 200 μg/ml could also remove most of living bacteria.

### Gene Expression Analysis

**FIGURE 8:**
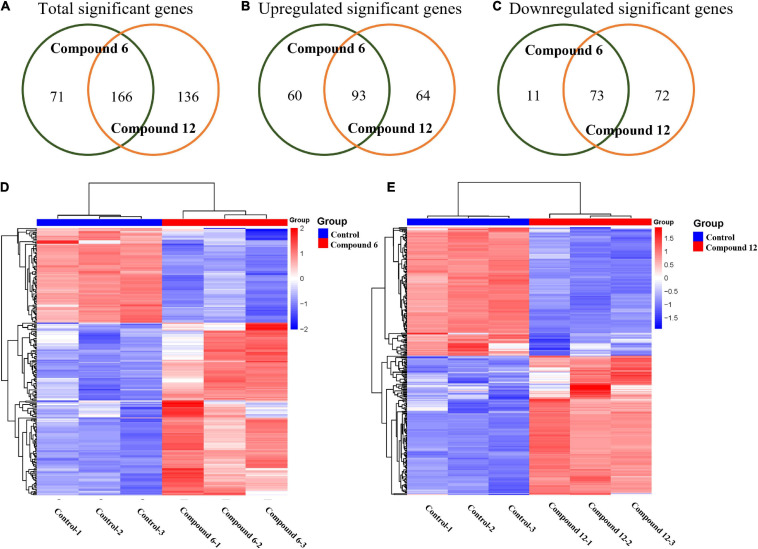
Gene expression analysis of MRSA biofilm cells treated with compounds **6** and **12** at concentration of 100 μg/ml. **(A–C)** Venn diagrams of total significant, upregulated, and downregulated significant genes in MRSA biofilm cells due to compounds **6** and **12** treatments. Heatmaps of the cluster analysis of differentially expressed genes between control group compared with compounds **6 (D)** and **12** treatment group **(E)**. Numbers 1–3 represent the three replicates. The normalized log-transformed counts are indicated by the color key. Red indicates upregulations, white represents intermediate expression, and blue indicates downregulations in the heatmaps.

All the sequencing reads of gene expression of MRSA cells under treatment with 100 μg/ml of compounds **6** and **12** have been submitted to the NCBI Gene Expression Omnibus (GEO) with accession number GSE147157. Overall, among 3,050 unigenes of the reference strain *S. aureus* A9754, 2,989 unigenes (98%) matched those in the NR database and 2,088 (68.46%) matched those in Swiss-Prot. After treatment with compounds **6** and **12**, 237 and 302 DEGs were detected compared with the control group, respectively (Figure 8A). A total of 84 and 153 genes were down- and up-regulated respectively in compound **6**-treated group, while there were 145 downregulated and 157 upregulated genes in compound **12**-treated group (Figures 8B,C). The Venn diagrams showed that 166 DEGs (including 73 downregulated and 93 upregulated) had the same expression trend in both treatment groups (Figures 8A–C). The heatmaps revealed the induced and suppressed transcripts in the treatment groups compared with the control group (Figures 8D,E).

### Functional Analyses of Differentially Expressed Genes

**FIGURE 9:**
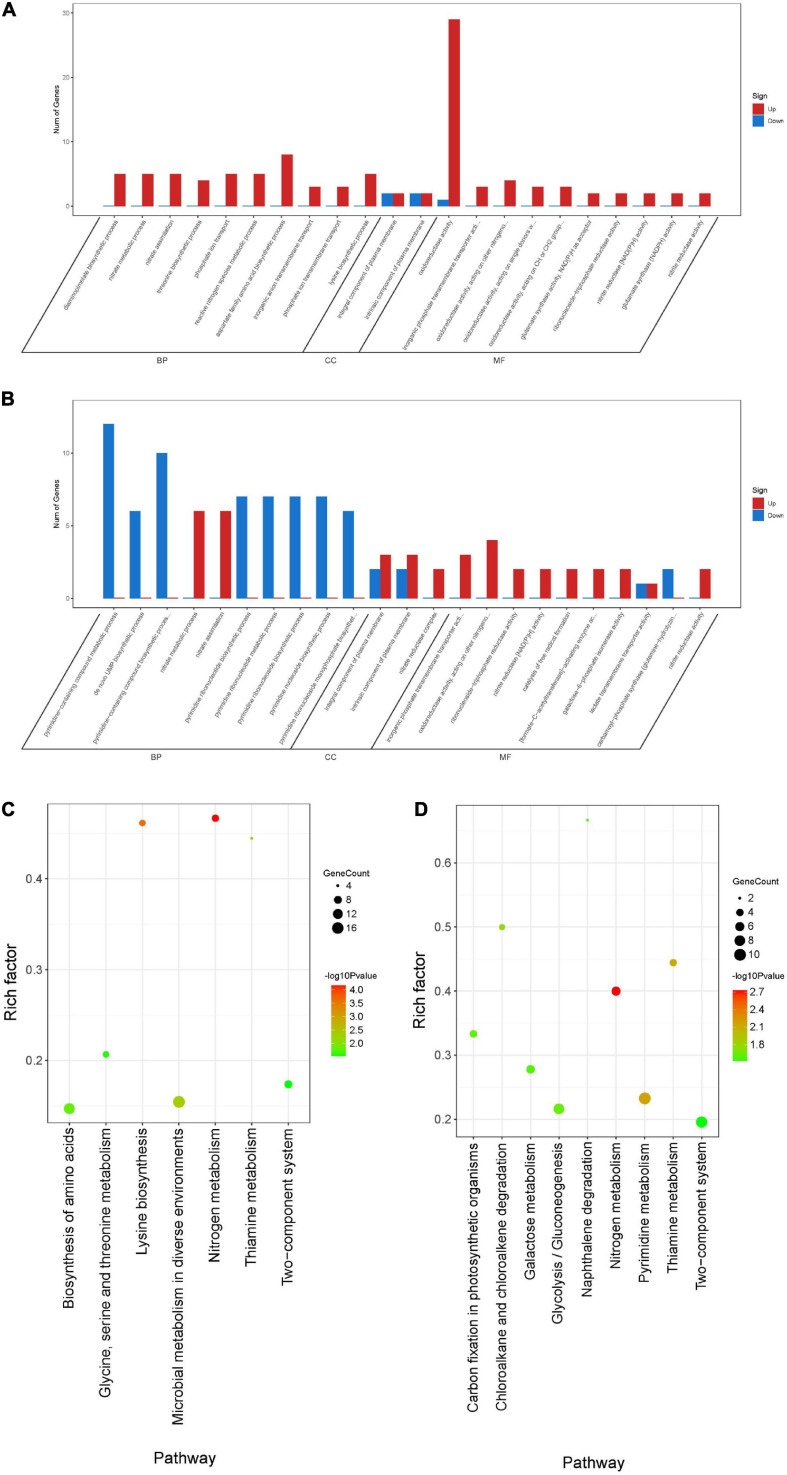
Differentially expressed genes annotated in GO terms and KEGG pathway. **(A,B)** Genes annotated in three main categories: biological process, cellular component, and molecular function after treatment with compounds **6 (A)** and **12 (B)**. **(C,D)** The KEGG pathway enrichment results of compounds **6 (C)** and **12 (D)** treatment groups. BP, biological process; CC, cellular component; MF, molecular function.

To further understand the function of DEGs, GO enrichment and KEGG pathway analyses of two treatment groups were performed respectively (Figure 9). According to GO classification analysis, DEGs were classified into three categories: biological process, cellular component, and molecular function. The GO enrichment analyses showed that the treatment groups had great different effects on the transcriptome of biofilm cells (Figures 9A,B). As in the biological process, all the GO term-related genes in the compound **6** treatment group were upregulated while only 2 out of 10 term-related genes were upregulated in the compound **12** treatment group. On the other hand, there were also similar changes in both treatment groups, such as the upregulation of nitrate metabolic process (GO: 0042126) and nitrate assimilation (GO: 0042128) in biological processes and the four terms enriched in molecular function were the same in both treatment groups. KEGG pathway analysis was used to clarify the biological function of genes (Figures 9C,D). A total of seven and nine pathways were significantly enriched in the compounds **6** and **12** treatment group, respectively. Among the pathway enrichment in the compound **6** treatment group, there were 17, 14, and 8 DEGs belonging to microbial metabolism in diverse environments, biosynthesis of amino acids, and two-component system, respectively. In the compound **12** treatment group, there were 10 DEGs that belonged to pyrimidine metabolism, 9 DEGs belonged to two-component system, and 8 DEGs belonged to glycolysis/gluconeogenesis pathway.

### Overlapped Differentially Expressed Genes of Two Treatments

**FIGURE 10:**
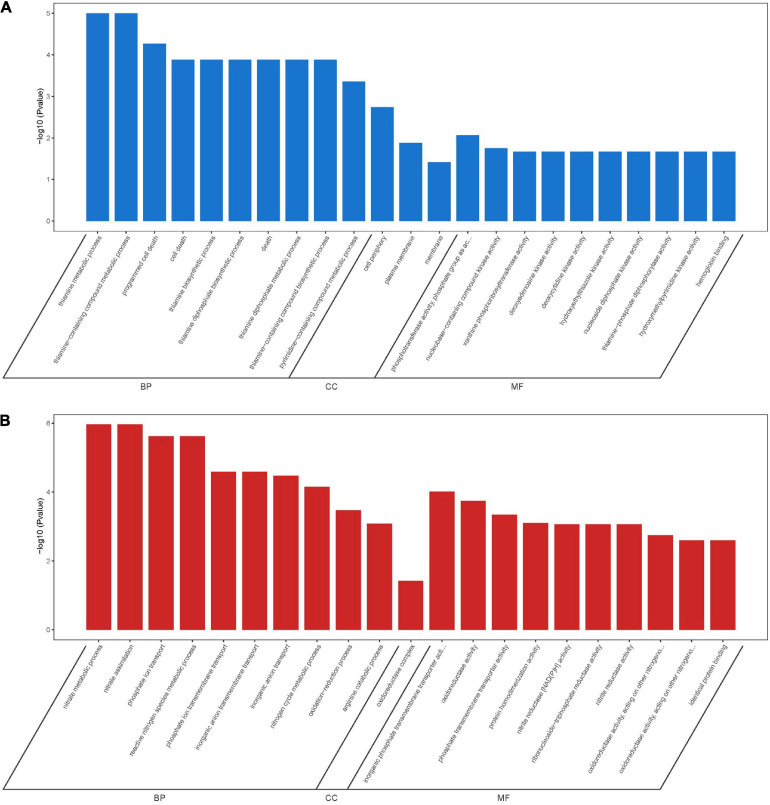
GO enrichment analysis of overlapping DEGs in both compounds **6** and **12** treatment groups. Significantly downregulated GO terms **(A)** are shown in blue, and significantly upregulated GO terms **(B)** are in red. BP, biological process; CC, cellular component; MF, molecular function.

A total of 166 DEGs, including 73 downregulated and 93 upregulated, overlapped in compounds **6** and **12** treatment groups. As shown in Figure 10A, the majority of downregulated DEGs were enriched to cellular component ontology, of which 26 genes were enriched in cell periphery (GO:0071944), 23 genes in plasma membrane (GO:0005886), and 23 genes in membrane (GO:0016020). The GO terms related to compound biosynthesis and metabolic processes were frequently shown in biological process category, and the kinase- and transferase-related terms were mainly enriched in molecular function category. Meanwhile, the upregulated categories (Figure 10B) showed that metabolic process- and carbolic process-related GO terms were prevalent in the biological process category, oxidoreductase activity, and binding and transporter activity in the molecular function category. Among these three categories, most of DEGs were enriched in biological process with 30 genes enriched in single-organism metabolic process (GO:0044710), followed by 22 genes in small molecule metabolic process (GO:0044281) and 16 genes in oxidation-reduction process (GO:0055114); 16 genes enriched in oxidoreductase activity (GO:0016491) were the largest group in molecular function category; oxidoreductase complex (GO:1990204) with two significant genes was annotated in cellular component category.

### qRT-PCR Validation

**FIGURE 11:**
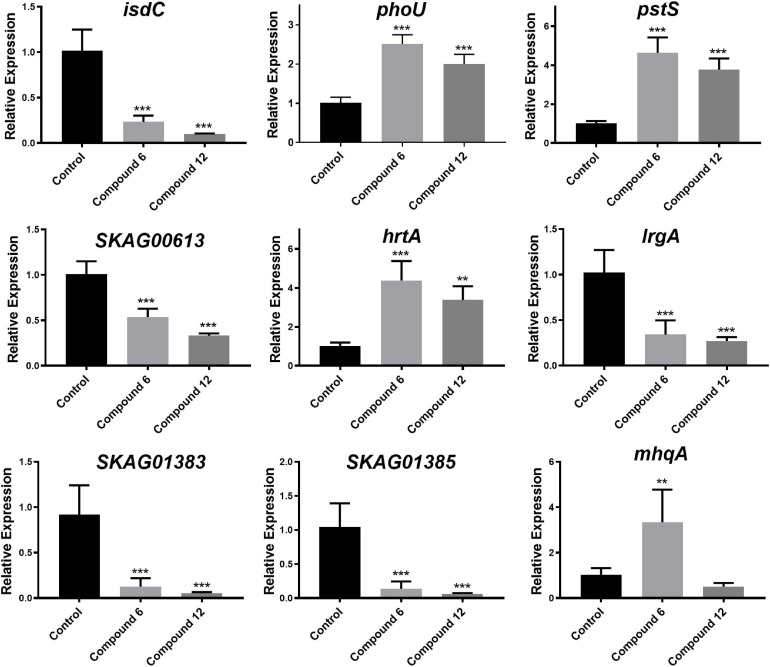
Relative expression of nine selected genes. The data represent mean ± SD (*n* = 4); ***p < 0.001, **p < 0.01.

To validate the RNA-Seq results, some genes involved in phosphate-specific transport system (*phoU* and *pstS*), iron-acquisition/transport (*isdC*, *SKAG_01383* and *SKAG_001385*), and other genes (*lrgA*, *mhqA*, *hrtA*, and *SKAG_00613*) were selected to conduct the qRT-PCR analysis (Figure 11 and Supplementary Tables 1, 2). The results showed that *isdC*, *lrgA*, *SKAG_00613*, *SKAG_01383*, and *SKAG_001385* genes were significantly downregulated in two groups. On the contrary, *phoU*, *pstS*, and *hrtA* were significantly upregulated in both groups. What is more, *mhqA* was significantly upregulated in compound **6** treatment group, while it had no significant change in compound **12** treatment group. In general, most of the qRT-PCR results of these selected genes were consistent with the RNA-Seq results, indicating that the RNA-Seq data were reliable.

## Discussion

In this study, a total of 10 structurally related AQs including two microbe-derived natural products DMCA (**1**) and TMCA (**2**) and their eight analogs were tested for antibiofilm activity. Among these compounds, only anthraquinone-2-carboxlic acid (**6**) and rhein (**12**) exhibited bactericidal activity with MIC values lower than 200 μg/ml. Both compounds had a carboxyl group attached to the C-2 position of the anthraquinone skeleton, which is different from other tested anthraquinones. For antibiofilm activity, some of the hydroxy-anthraquinones have been reported for their biofilm-inhibiting activity, and previous SAR analysis suggested that the hydroxyl groups at the C-1 and C-2 positions of the anthraquinone skeleton should play an important role in inhibiting biofilm formation at low concentrations (lower than 10 μg/ml) (Manoharan et al., 2017). Herein, it is interesting to note that the single hydroxyl group at the C-1 and C-2 positions exerted different biofilm inhibition activities at high concentrations (50 and 200 μg/ml). When comparing compounds **3**–**5**, compound **5** showed a higher rate of biofilm inhibition than compounds **3** and **4**, suggesting that the substitution of a hydroxyl group at the C-2 position was more important than the C-1 position in enhancing the prevention capability of MRSA biofilm formation. This was also supported by the good biofilm inhibitory activity of compounds **8** and **9**, both of which also have a C-2 hydroxyl group. Surprisingly, compound **7** with two symmetric hydroxyl groups at C-2 promoted biofilm formation instead, which was different from all the other compounds and requires further investigation. Consistent with the literature (Manoharan et al., 2017), at high concentrations, compounds **8**, **9**, and **11** still had excellent inhibitory performance for MRSA biofilm, but compounds **3**, **4**, **7**, and **10** could not suppress biofilm formation.

The biofilm eradication activity of these hydroxy- and carboxy-substituted AQs was then analyzed, and the SAR study revealed that: (i) for the hydroxyl AQs **5**, **7**, and **9**, the biofilm eradication rates of these three compounds were all around 20–30% at the concentration of 200 μg/ml, suggesting that the number of hydroxyl substitutions might not have much correlation with their activity; (ii) for the carboxylic AQs **3**, **6**, and **12**, compounds **6** and **12** showed higher biofilm eradication efficiency (the respective eradication rate of 54.2 ± 5.9 and 44.0 ± 5.3% at 200 μg/ml, and 57.7 ± 3.8 and 26.5 ± 3.2% at 50 μg/ml for compounds **6** and **12**, respectively) than that of compound **3**, indicating that the carboxyl group at C-2 position of the anthraquinone skeleton might be very important for improving eradication activity against MRSA biofilm; and (iii) combining the results of all the SAR analysis of hydroxy- and carboxy-AQs, we could conclude that the AQs including the carboxyl group at C-2 position of anthraquinone mother nucleus exhibited better MRSA biofilm eradication activity than the hydroxyl ones. Therefore, we suggest that the carboxyl substitution attached at C-2 position of anthraquinone should be considered a key group for further chemical design and development of antibiofilm AQs. In addition, it is particularly noteworthy that the biofilm eradication activity of carboxyl-substituted anthraquinone compounds **6** and **12** was reported for the first time in this study.

Since compounds **6** and **12** displayed promising eradication activity against MRSA biofilm, different staining methods, microscopy images, and RNA-Seq analysis were employed for more in-depth study. It was found that the MTT staining test always displayed a higher biofilm eradication rate than the CV staining test under the same concentrations of both compounds, which lead us to wonder whether their biofilm eradication effects against the preformed MRSA biofilms mostly involved decreasing the viable cells within biofilms rather than reducing the total biomass. Thus, the SEM imaging of the biofilm structures treated with these two compounds was carried out, and results showed visibly structural disruption of the treated biofilms. And the CLSM imaging analysis revealed that a significant decrease of the living biofilm cells in both treatment groups. All these results indicated that compounds **6** and **12** could gently reduce the total biofilm biomass but effectively remove the viable biofilm cells when exerting their antibiofilm activity.

For RNA-Seq analysis, we focused on the comparison of our transcriptomic results with those reported in literature (Monds et al., 2001; O’May et al., 2009; Neznansky and Opatowsky, 2014; Wang et al., 2017; Abouelhassan et al., 2018) due to the currently ambiguous and incomplete mechanism of the MRSA biofilm eradication. Recently, a halogenated phenazine has been reported to eradicate MRSA biofilms depending on an iron-starvation mechanism (Abouelhassan et al., 2018). The RNA-Seq results of compounds **6** and **12** treatment groups showed that most of the iron-acquisition– and iron-transport–related genes (*isdABCEFGHI*, *sbnAEI*, and *mntAB*) were downregulated or insignificantly differentially expressed instead of upregulated (Supplementary Table 2). Even though the iron ion might not be involved with the working mechanism of our compounds, it provided us a clue to analyze other ions. Meanwhile, among the overlapped DEGs of the two experimental groups, except for those involved in metabolic- and catabolic-related progress or function, many of the other upregulated genes belonged to transport GO terms: anion transport (GO:0006820), inorganic phosphate transmembrane transporter activity (GO:0005315), and phosphate transmembrane transporter activity (GO:1901677). It has been reported that the phosphate-specific transport system (Pst system; Supplementary Table 2), including five genes *pstSCAB-phoU*, has a high affinity for uptake of inorganic phosphate (P_*i*_) and is very important for cell growth. What is more, the *pst* mutants revealed that these genes are necessary for the biofilm formation of different bacteria (Monds et al., 2001; O’May et al., 2009; Neznansky and Opatowsky, 2014; Wang et al., 2017). In our study, the upregulation of these genes suggested that P_*i*_ uptake was increased after treatment of compound **6** or **12**. The redundant P_*i*_ may be used to synthesize high-molecular-weight inorganic polyphosphate to store energy for cell metabolism and other purpose (Monds et al., 2001; Wang et al., 2017). We speculated that compounds **6** and **12** might influence phosphate homeostasis in MRSA which in turn led to the disassembly of biofilms, and more experiments are required to confirm this hypothesis.

As for the safety of compounds **6** and **12**, numerous studies have been carried out to study their biological activities and related molecular mechanisms (Sheng et al., 2011; Zhao et al., 2011; KoraMagazi et al., 2016; Panigrahi et al., 2016; Park et al., 2016a, b; Yuan et al., 2016). Take the cytotoxicity for instance, compound **6** had been demonstrated to have anti-inflammatory activity without cellular toxicity at concentrations below 26.8 μg/ml (Park et al., 2016a, b). On the contrary, compound **12**, which has been well studied in liver diseases, could trigger liver cytotoxicity in the long-term use, and the cytotoxicity could take place quickly when used over 2.8 μg/ml (Sheng et al., 2011; Zhao et al., 2011; KoraMagazi et al., 2016; Panigrahi et al., 2016; Yuan et al., 2016). Herein, although both compounds displayed biofilm eradication activity at the lowest effective concentration of 12.5 μg/ml, it seems that compound **6** might be safer than compound **12** when used as an antibiofilm agent.

In our study, the antibacterial and antibiofilm activities against MRSA of a series of structurally related AQs have been well evaluated. The SAR analysis demonstrated that different positions of carboxyl and hydroxyl substitutions of the anthraquinone skeleton can significantly affect antibiofilm activity of these compounds. A hydroxyl group at the C-2 position of the anthraquinone skeleton was much more important than the C-1 position for improving their inhibitory activity against MRSA biofilm formation. Carboxylic AQs were more effective in the eradication of MRSA biofilms than the hydroxyl ones. The results of MTT staining and CV staining test, SEM and CLSM images, and RNA sequencing revealed that the MRSA biofilm eradication mechanism of compounds **6** and **12** might involve dispersion of biofilm cells and disruption of phosphate homeostasis.

## Data Availability Statement

The datasets presented in this study can be found in online repositories. The names of the repository/repositories and accession number(s) can be found in the article/Supplementary Material.

## Author Contributions

Z-MS and YX designed the project. Z-MS and K-LW carried out experiments, interpreted the results, and wrote the manuscript. K-LW, YX, and C-YW funded the research and revised the manuscript. KZ, L-MY, and YZ helped in the RNA-Seq analysis. J-LZ provided a lot of help during the experiment. All authors contributed to the article and approved the submitted version.

## Conflict of Interest

The authors declare that the research was conducted in the absence of any commercial or financial relationships that could be construed as a potential conflict of interest.

## Publisher’s Note

All claims expressed in this article are solely those of the authors and do not necessarily represent those of their affiliated organizations, or those of the publisher, the editors and the reviewers. Any product that may be evaluated in this article, or claim that may be made by its manufacturer, is not guaranteed or endorsed by the publisher.

## Footnotes

1
http://www.bioinformatics.babraham.ac.uk/projects/fastqc/

2
http://bacteria.ensembl.org/

3
https://cran.r-project.org/doc/FAQ/R-FAQ.html#Citing-R
