# Supplementary material for: Anthraquinones as Potential Antibiofilm Agents Against Methicillin-Resistant Staphylococcus aureus
Source: Front Microbiol. 2021 Sep 3;12:709826. doi: 10.3389/fmicb.2021.709826 (PMC8446625; doi:10.3389/fmicb.2021.709826)
Supplement: Supplementary file 1 [file Data_Sheet_1.docx]

**Anthraquinones as potential anti-biofilm agents against** **methicillin-resistant *Staphylococcus aureus***

**Zhi-Man Song^1,2,3^, Jun-Liang Zhang^1^, Kun Zhou^1^, Lu-Ming Yue^1^, Yu Zhang^1^, Chang-Yun Wang^4,5,6^, Kai-Ling Wang^3^* and Ying Xu^1^***

^1^Shenzhen Key Laboratory of Marine Bioresource and Eco-Environmental Science, Shenzhen Engineering Laboratory for Marine Algal Biotechnology, College of Life Sciences and Oceanography, Shenzhen University, Shenzhen, China.

^2^Department of Chemistry, The University of Hong Kong, Pokfulam, Hong Kong, China.

^3^College of Pharmacy, Institute of Materia Medica, Dali University, Dali, China.

^4^Key Laboratory of Marine Drugs, The Ministry of Education of China, School of Medicine and Pharmacy, Ocean University of China, Qingdao, China.

^5^Laboratory for Marine Drugs and Bioproducts, Qingdao National Laboratory for Marine Science and Technology, Qingdao, China.

^6^Institute of Evolution and Marine Biodiversity, Ocean University of China, Qingdao, China.

***Corresponding authors:** Kai-Ling Wang and Ying Xu

email: [kailingw@dali.edu.cn](mailto:kailingw@dali.edu.cn), [boxuying@szu.edu.cn](mailto:boxuying@szu.edu.cn)

**
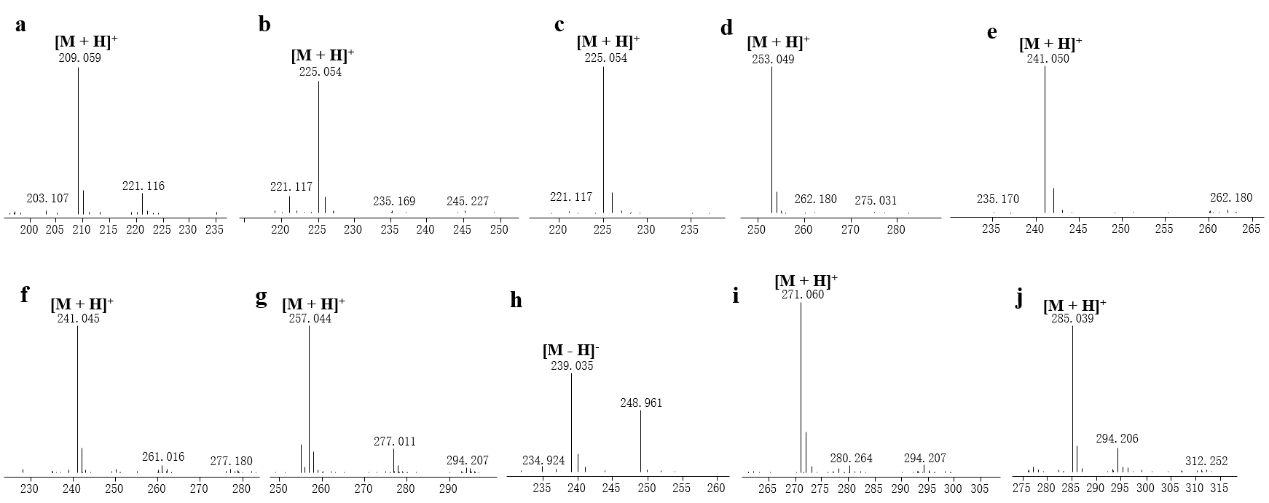
**

Fig S1. LC-MS data of commercially obtained anthraquinones. **a**-**j** represents for compound **3**-**12**.

Fig S2. ^1^H NMR spectrum of compound **1** in DMSO-*d*_6_

Fig S3. ^13^C NMR spectrum of compound **1** in DMSO-*d*_6_.


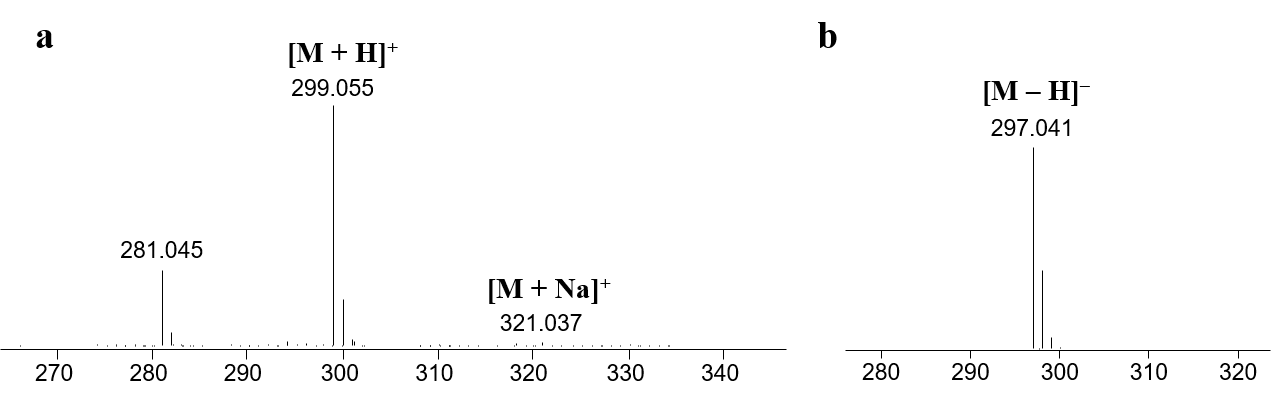
Fig S4. LC-MS date of compound **1**.

Fig S5. ^1^H NMR spectrum of compound **2** in DMSO-*d*_6_

**
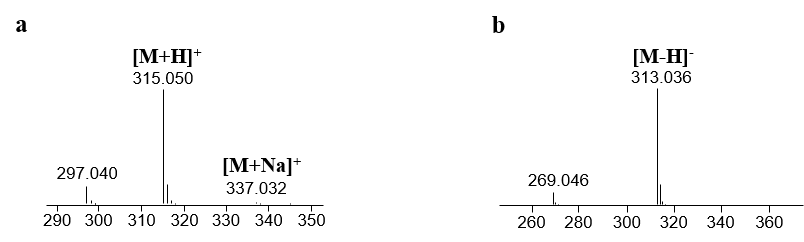
**

Fig S6. LC-MS data of compound **2**.

Table S1. Primers used in real- time PCR.

| Gene ID | Primer sequence（5’-3’） |
| --- | --- |
| *pyk*-F | GCATCTGTACTCTTACGTCC |
| *pyk*-R | GGTGACTCCAAGTGAAGA |
| *SKAG_00180*-F | CATCATCGCGACATTCAGT |
| *SKAG_00180*-R | TCCAATGACTGTGGTTGACA |
| *SKAG_00452*-F | CAGCGACAAATGGATCGTTA |
| *SKAG_00452*-R | ACCCGTTTAAAGACAATGGG |
| *SKAG_00456*-F | CTATTTTAAGTTGATAAGCTTGTAG |
| *SKAG_00456*-R | TGAAACATGATTTTGATGGTGAA |
| *SKAG_00613*-F | ATCTTCACCTTCACGTTTCA |
| *SKAG_00613*-R | GTTATTCCTTCCACACCGTA |
| *SKAG_00974*-F | TTTGTTGTTTGGTCATTCCC |
| *SKAG_00974*-R | CAGCATCGTCCTAGTGATTT |
| *SKAG_01273*-F | TGCCTGCATCAGTAATCGGT |
| *SKAG_01273*-R | ATACCGGCTGGTACGAAGAG |
| *SKAG_01383*-F | TAGCCTTATTACTTCTAGTTGC |
| *SKAG_01383*-R | TATCGACGTTGTCTCCAC |
| *SKAG_01385*-F | AGGTCGTCTAAACCATCCAA |
| *SKAG_01385*-R | CCAGTACGCGGCGAAATAAT |
| *SKAG_01708*-F | GGCCTAATCCATAAATCGCT |
| *SKAG_01708*-R | GTGTTGCCATTTGAAGAAGT |
